# McKusick-Kaufman Syndrome: A Case Report With an Emphasis on Perinatal Diagnosis and Genetic Counseling

**DOI:** 10.7759/cureus.37808

**Published:** 2023-04-19

**Authors:** Sankalp Khanke, Aman Agrawal, Vaishnavi Toshniwal, Sanket S Bakshi, Aruna Chandak

**Affiliations:** 1 Medical School, Jawaharlal Nehru Medical College, Datta Meghe Institute of Higher Education and Research, Wardha, IND; 2 Anaesthesiology, Jawaharlal Nehru Medical College, Datta Meghe Institute of Higher Education and Research, Wardha, IND

**Keywords:** polydactyly, hydrometrocolpos, chromosome 20, mkks gene, mckusick–kaufman syndrome

## Abstract

McKusick-Kaufman syndrome is a rare genetic disorder that affects limb development, genital formation, and heart function. It is caused by mutations in the *MKKS* gene on chromosome 20. Individuals with this condition may have extra fingers or toes, fused labia or undescended testes, and, less commonly, severe heart defects. Diagnosis involves a physical examination and genetic testing, while treatment focuses on symptom management, including surgical intervention if necessary. The prognosis varies depending on the severity of associated complications. In a recent case, a 27-year-old woman with fetal hydrometrocolpos gave birth to a female neonate with extra digits on both hands and feet, fused labia, and a small vaginal opening. The neonate also had a large abdominal cystic mass, and echocardiography revealed a patent foramen ovale. Genetic testing confirmed an *MKKS *gene mutation, and the hydrometrocolpos required surgical management. Early diagnosis and intervention can improve outcomes for individuals with this syndrome.

## Introduction

McKusick-Kaufman syndrome (MKS) is an autosomal recessive condition that includes genital malformations, limb abnormalities, and heart defects [1]. The condition is caused by a mutation on chromosome 20 in the *MKKS *gene [2]. These specific genes play a critical role in the development and maintenance of cilia, which are tiny hair-like projections that cover the surface of various types of cells. Cilia are involved in numerous biological processes and are essential for their proper functioning. The condition was first described by Victor McKusick and Robert Kaufman in 1970, and since then, fewer than 100 cases have been reported worldwide [3]. The limb abnormalities usually involve extra fingers or toes, and the genital malformations may include fused labia or undescended testes. Heart defects are less common but can be severe. MKS is a genetic disorder that primarily affects females and is characterized by an abnormality in the genitalia called hydrometrocolpos. Hydrometrocolpos is a condition where there is a buildup of fluid in the pelvis due to a blockage in the vagina that occurs before birth. This blockage can be caused by incomplete development of the vagina (vaginal agenesis) or by a membrane blocking the vaginal opening. Diagnosis is based on physical examination and genetic testing. Treatment involves the management of specific symptoms and may include surgery. The prognosis varies depending on the severity of the heart defects and other associated complications. Early diagnosis and intervention can improve outcomes for affected individuals [4].

## Case presentation

**Figure 1:**
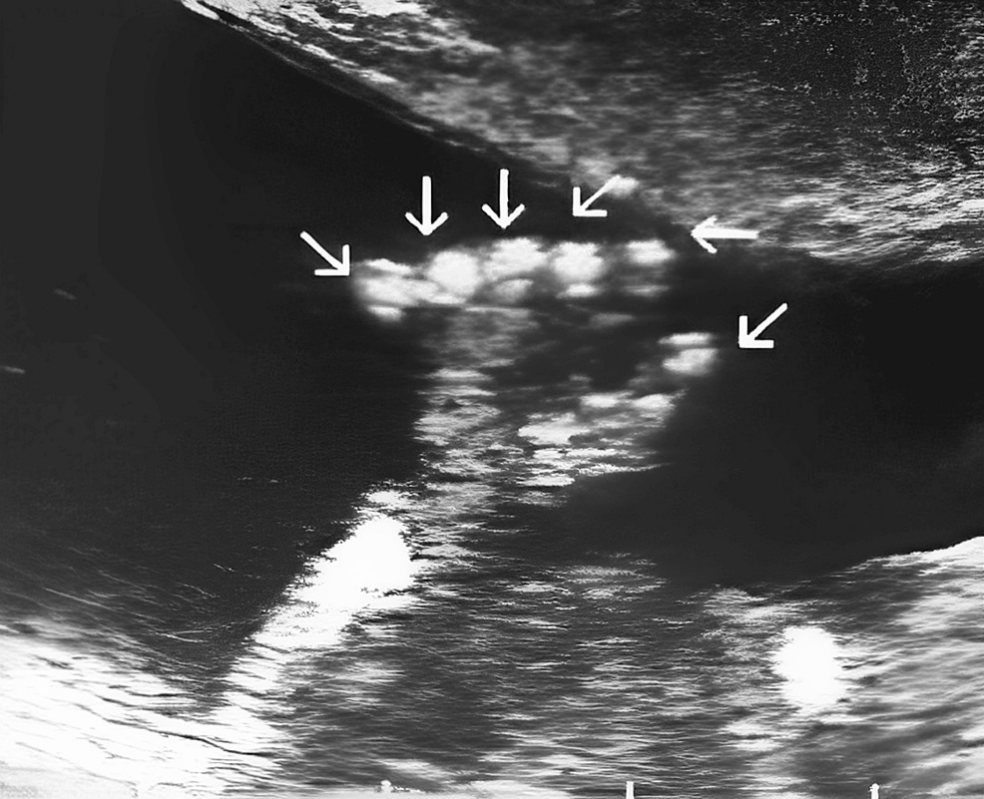
Level II ultrasound scan showing polydactyly.

A 27-year-old pregnant woman visited the antenatal clinic at 28 weeks of gestation with an obstetric score of G5P4A0. She had no significant past medical or surgical history. An ultrasound scan was conducted which showed a single viable fetus with polydactyly in both hands and a large fluid-filled structure in the fetal pelvis. The amniotic fluid volume was normal, and no other structural abnormalities were detected. A referral was made for a level II ultrasound scan, confirming polydactyly (Figure 1).

There were no other anomalies detected, and the fetal heart was normal.

Based on the ultrasound findings, fetal hydrometrocolpos was diagnosed, and the patient was informed about the possibility of an underlying genetic disorder. Amniocentesis was offered, but the patient declined. The patient was managed conservatively with regular ultrasound monitoring of fetal growth and amniotic fluid volume.

At 39 weeks of gestation, the patient presented in spontaneous labor, and a female neonate was delivered via cesarean section with an APGAR score of eight at one and five minutes. As mentioned above, the patient had a history of four pregnancies and deliveries, with no abortions or any other medical interruptions of pregnancy. Her prenatal history and laboratory reports were normal. At 35 weeks of gestation, an ultrasound showed an abdominal mass and a dilated lateral ventricle of the brain.

**Figure 2:**
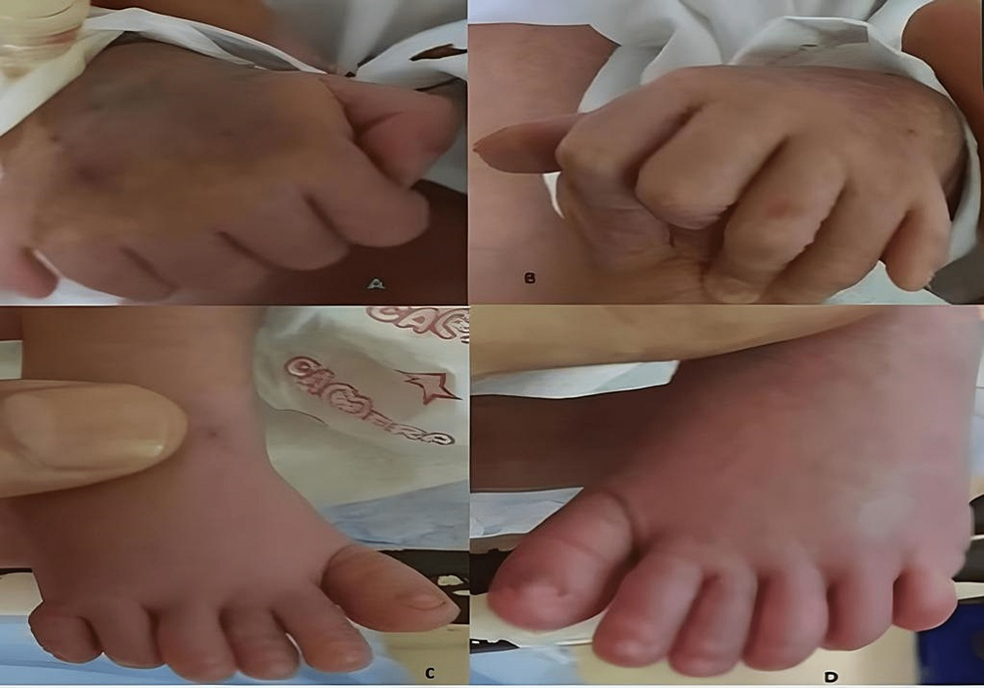
(A) Polydactyly in the right upper limb. (B) Polydactyly in the left upper limb. (C) Polydactyly in the right lower limb. (D) Polydactyly in the left lower limb.

**Figure 3:**
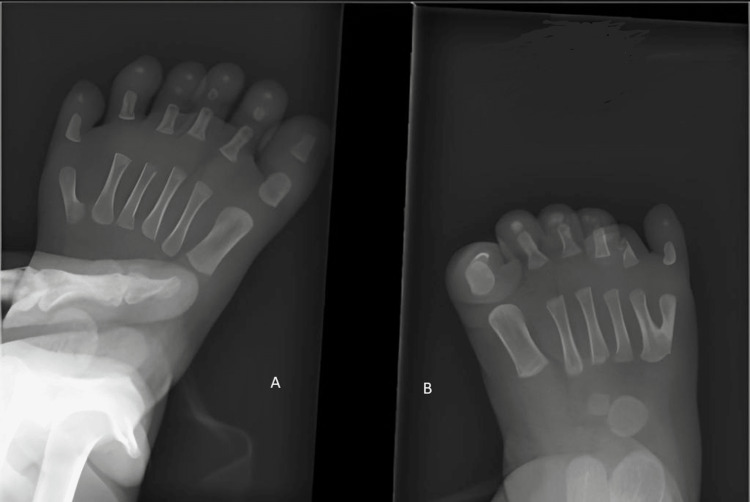
(A) X-ray of the left foot showing polydactyly. (B) X-ray of the right foot showing polydactyly.

The postnatal examination showed that the baby was 44 cm long, with a head circumference of 35 cm and a weight of 2.8 kg. The baby had respiratory distress and required 2 L/minute of nasal oxygen. Physical examination revealed the presence of an extra digit on both hands and feet, giving her a total of 12 fingers and 12 toes. The extra digits were well-formed and had functional joints (Figures 2, 3).

**Figure 4:**
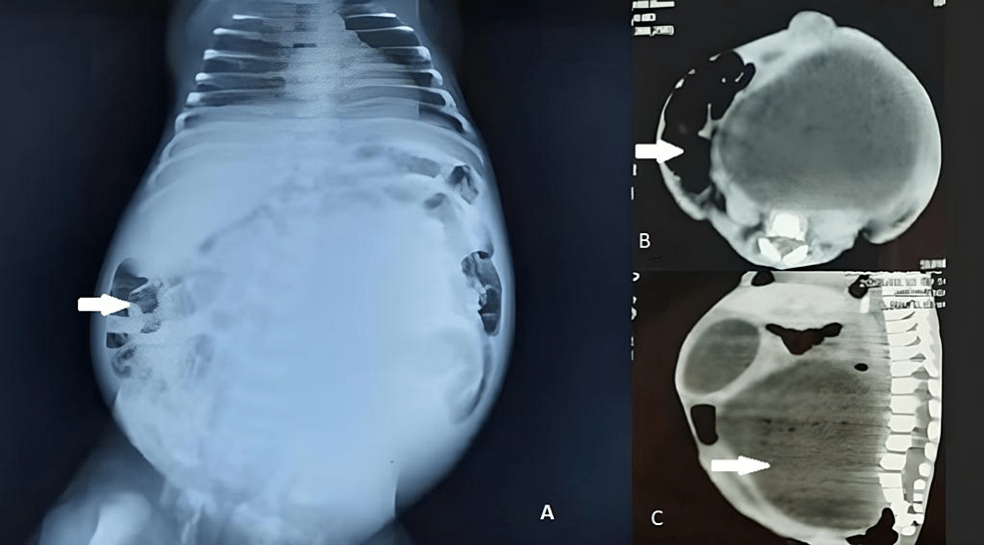
(A) X-ray of the abdomen. (B, C) CT scan showing hydrometrocolpos.

On abdominal examination, her abdomen was found to be distended. Additionally, the baby had fused labia and a small vaginal opening. There was no evidence of clitoromegaly, and her external genitalia appeared otherwise normal. A cardiac examination did not reveal any murmurs or abnormal sounds. Abdominal ultrasound and renal function tests were unremarkable. A large fixed cystic mass was present on the abdominal examination, which was confirmed on a CT scan and X-ray of the abdomen (Figure 4).

The baby also showed bilateral pes cavus and polydactyly in her upper and lower limbs. Cystoscopy and genitography revealed the presence of hydrometrocolpos and a transverse vaginal septum. Echocardiography was advised, which was suggestive of a patent foramen ovale. Additionally, next-generation sequencing revealed an *MKKS *gene mutation.

The hydrometrocolpos required surgical management, and the patient was referred to a pediatric surgeon. To assess the condition, a cystoscopy and vaginoscopy were conducted, and a Foley catheter was inserted into the bladder. An incision was made in the vaginal dimple, and a significant quantity of fluid was drained. The vaginal dimple was then expanded, and a Foley catheter was placed in the vaginal vault to maintain its openness. The patient received prophylactic antibiotics, and after 48 hours, the Foley catheter was removed. An additional 1.2 L of fluid was aspirated, which improved the respiratory effort of the newborn. The patient was discharged after five days and did well on follow-up.

## Discussion

MKS is a rare autosomal recessive genetic disorder with a prevalence rate of 1 in 10,000 individuals. This syndrome is characterized by extra fingers or toes (polydactyly), genital malformations, and heart defects. It was first described in 1964 by Victor A. McKusick and Robert L. Kaufman. MKS is caused by mutations in the *MKKS *gene located on chromosome 20p12.2-p12.1 [4].

The most common limb abnormality observed in MKS is polydactyly, ranging from a small extra digit to a fully formed finger or toe. Genital malformations in females typically involve fused labia and a small vaginal opening, while males may have undescended testes. Heart defects, although less common, can be severe and may require surgical intervention [5].

We can contemplate a differential diagnosis of MKS and Bardet-Biedl syndrome (BBS) because genital abnormalities and limb abnormalities are commonly present in both disease entities [6]. BBS is another autosomal recessive disorder that shares some clinical features with MKS, including limb abnormalities and genital malformations. However, BBS is distinguished by central obesity, learning disabilities, retinitis pigmentosa, renal anomalies, and hypogonadism [7-10].

Diagnosis of MKS is typically based on physical examination and genetic testing. Individuals with MKS often exhibit multiple physical abnormalities, including polydactyly, genital malformations, and heart defects. Genetic testing can confirm the presence of mutations in the *MKKS *gene, which is diagnostic for MKS [4].

Management of MKS involves a multidisciplinary approach, with treatment aimed at managing the specific symptoms of the condition. Surgery may be recommended to correct genital malformations and prevent long-term complications. Individuals with congenital heart defects may require cardiac surgery to correct the abnormalities [11].

The prognosis for individuals with MKS varies depending on the severity of their symptoms and the presence of associated complications. Early diagnosis and intervention can improve outcomes for affected individuals, highlighting the importance of timely identification and collaborative treatment. Recent advances in medical technology and research have shown promise in improving the management of MKS. For example, targeted therapies and gene therapies have the potential to address the underlying genetic mutations that cause the condition [12].

In essence, this case offers a thorough examination of MKS and stresses the significance of genetic testing which can aid in early diagnosis and collaborative treatment. Because this is a rare condition, very few cases have been published and very few studies have been done. It would be helpful if more articles or cases would have been discussed to gain more information and emphasize the importance of further investigation and effective treatments. Additionally, the study highlights the possible benefits of novel treatments and the necessity of further investigation and medical attention to enhance the quality of life for people impacted by this uncommon condition.

## Conclusions

MKS is an autosomal recessive condition distinguished by limb abnormalities, genital malformations, and heart defects. The prevalence of this disorder is about 1 in 14,000 individuals. The diagnosis is based on genetic testing and physical examination. Treatment requires a multidisciplinary approach aimed at managing specific symptoms. The prognosis varies depending on the severity of the heart defects and other associated complications. Early diagnosis and intervention can improve outcomes for affected individuals. This case report offers a thorough analysis of MKS and emphasizes the importance of further investigation and medical attention to enhance the quality of life for individuals impacted by this infrequent condition.

## References

[1] Adam A, Hellig J, Mahomed N, Lambie L (2017). Recurrent urinary tract infections in a female child with polydactyly and a pelvic mass: consider the McKusick-Kaufman syndrome. Urology.

[2] Katsanis N, Beales PL, Woods MO (2000). Mutations in MKKS cause obesity, retinal dystrophy and renal malformations associated with Bardet-Biedl syndrome. Nat Genet.

[3] B Hatti R, V Badakali A, N Vanaki R, S Samalad M (2013). Mckusick-Kaufman syndrome presenting as acute intestinal obstruction. J Neonatal Surg.

[4] Ullah I, Rauf S, Ali S (2022). A case of McKusick-Kaufman syndrome with perinatal diagnosis: case report and literature review. Ann Med Surg (Lond).

[5] Stone DL, Agarwala R, Schäffer AA (1998). Genetic and physical mapping of the McKusick-Kaufman syndrome. Hum Mol Genet.

[6] Slavotinek AM, Biesecker LG (2000). Phenotypic overlap of McKusick-Kaufman syndrome with Bardet-Biedl syndrome: a literature review. Am J Med Genet.

[7] Klein D, Ammann F (1969). The syndrome of Laurence-Moon-Bardet-Biedl and allied diseases in Switzerland. Clinical, genetic and epidemiological studies. J Neurol Sci.

[8] Schachat AP, Maumenee IH (1982). Bardet-Biedl syndrome and related disorders. Arch Ophthalmol.

[9] Green JS, Parfrey PS, Harnett JD (1989). The cardinal manifestations of Bardet-Biedl syndrome, a form of Laurence-Moon-Biedl syndrome. N Engl J Med.

[10] Beales PL, Elcioglu N, Woolf AS, Parker D, Flinter FA (1999). New criteria for improved diagnosis of Bardet-Biedl syndrome: results of a population survey. J Med Genet.

[11] Chitayat D, Hahm SY, Marion RW (1987). Further delineation of the McKusick-Kaufman hydrometrocolpos-polydactyly syndrome. Am J Dis Child.

[12] Schaefer E, Durand M, Stoetzel C (2011). Molecular diagnosis reveals genetic heterogeneity for the overlapping MKKS and BBS phenotypes. Eur J Med Genet.

